# Nutrient intake and risk of multimorbidity: a prospective cohort study of 25,389 women

**DOI:** 10.1186/s12889-024-18191-9

**Published:** 2024-03-04

**Authors:** Ge Song, Weimin Li, Yanfen Ma, Yao Xian, Xia Liao, Xueliang Yang, Huifeng Zhang, Janet E Cade

**Affiliations:** 1 Department of Clinical Nutrition, The First Affiliated Hospital of Xi’an Jiaotong University, No. 277 Yanta West Road, 710061 Xi’an, China; 2 Department of Clinical Laboratory, The First Affiliated Hospital of Xi’an Jiaotong University, No. 277 Yanta West Road, 710061 Xi’an, China; 3 School of Food Science and Nutrition, University of Leeds, LS2 9AT Leeds, UK

**Keywords:** Multimorbidity, Nutrient intake, Charlson comorbidity index, Hospital episode statistics

## Abstract

**Background:**

Multimorbidity is becoming an increasingly serious public health challenge in the aging population. The impact of nutrients on multimorbidity remains to be determined and was explored using data from a UK cohort study.

**Method:**

Our research analysis is mainly based on the data collected by the United Kingdom Women’s Cohort Study (UKWCS), which recruited 35,372 women aged 35–69 years at baseline (1995 to 1998), aiming to explore potential associations between diet and chronic diseases. Daily intakes of energy and nutrients were estimated using a validated 217-item food frequency questionnaire at recruitment. Multimorbidity was assessed using the Charlson comorbidity index (CCI) through electronic linkages to Hospital Episode Statistics up to March 2019. Cox’s proportional hazards models were used to estimate associations between daily intakes of nutrients and risk of multimorbidity. Those associations were also analyzed in multinomial logistic regression as a sensitivity analysis. In addition, a stratified analysis was conducted with age 60 as the cutoff point.

**Results:**

Among the 25,389 participants, 7,799 subjects (30.7%) were confirmed with multimorbidity over a median follow-up of 22 years. Compared with the lowest quintile, the highest quintile of daily intakes of energy and protein were associated with 8% and 12% increased risk of multimorbidity respectively (HR 1.08 (95% CI 1.01, 1.16), *p*-linearity = 0.022 for energy; 1.12 (1.04, 1.21), *p*-linearity = 0.003 for protein). Higher quintiles of daily intakes of vitamin C and iron had a slightly lowered risk of multimorbidity, compared to the lowest quintile. A significantly higher risk of multimorbidity was found to be linearly associated with higher intake quintiles of vitamin B12 and vitamin D (*p*-linearity = 0.001 and 0.002, respectively) in Cox models, which became insignificant in multinomial logistic regression. There was some evidence of effect modification by age in intakes of iron and vitamin B1 associated with the risk of multimorbidity (*p*-interaction = 0.006 and 0.025, respectively).

**Conclusions:**

Our findings highlight a link between nutrient intake and multimorbidity risk. However, there is uncertainty in our results, and more research is needed before definite conclusions can be reached.

**Supplementary Information:**

The online version contains supplementary material available at 10.1186/s12889-024-18191-9.

## Introduction

Multimorbidity, commonly defined as the coexistence of two or more chronic conditions within an individual [1, 2], is becoming one of the major challenges for health systems worldwide. According to a review including 41 articles, the prevalence of multimorbidity accounts for 20–30% of the whole population and 55–98% when the elderly are considered globally [3]. In the United States, a national survey showed that 59.6% of adults had multimorbidity in 2014 [4]. The proportion of patients with four + diseases is expected to double between 2015 and 2035 in the United Kingdom [5]. A large epidemiological survey in China found that 46.5% of residents had several chronic diseases as multimorbidity [6]. The prevalence is higher among very elderly people, women, and people with lower social classes [3, 4]. Multimorbidity is highly associated with increased risks of premature death, hospitalization, loss of physical functioning, depression, polypharmacy, and worsening quality of life, thus resulting in a substantial economic burden on health systems [7]. Multimorbidity patients had a 73% (HR: 1.73, (95% CI: 1.41, 2.13)) [8] increased risk of death and a 94% (1.94, (1.43, 2.63)) [9] increased risk of functional limitation compared with non-multimorbidity patients.

Several modifiable lifestyles, such as smoking, long-term sitting and obesity, are found to be risk factors for multimorbidity [10, 11]. The promotion of healthy behavior, especially adherence to a healthy diet, has been shown to be effective for preventing multimorbidity [12]. Currently, research on the relationship between nutrition (such as dietary patterns, consumed main foods, nutrient intake) and multimorbidity has been reported in several studies. A cross-sectional exploratory study from China reported that unhealthy dietary behaviors such as over-eating and intra-meal water drinking were linked to an increased risk of cardiometabolic multimorbidity [13]. Another cross-sectional study conducted in the Netherlands found that adults with cardiometabolic multimorbidity consumed more meat and snacks [14]. According to a Chinese cohort study, greater consumption of fruits, vegetables, and whole grain products appears to lower the risk of multimorbidity [15]. It has been reported that higher adherence to the Mediterranean diet was associated with a lower risk of multimorbidity [16, 17], and the Mediterranean diet may contribute to protecting geriatric patients with multimorbidity from developing depressive symptoms [18]. Yue Zhang et al. [19] found that a white meat pattern characterized by consumption of fish and poultry is associated with reduced risks of multimorbidity, while more frequent intake of poultry, the specific food group, was related to higher risks. Generally, most present evidence remains cross-sectional, and the number of cohorts is still limited, especially regarding nutrient intake. A cross-sectional study from South Korea found that calcium and potassium were associated with a lower cardiometabolic multimorbidity pattern [20]. Another cross-sectional study reported that lutein and zeaxanthin intake may be good for one’s health, whereas increasing niacin intake may be likely to raise 10-year mortality according to the Charlson comorbidity index (CCI) [21]. At present, the conclusions are still limited, and more high-quality longitudinal studies need to be conducted.

The aim of this study was to investigate the relationship between nutrients and multimorbidity. Knowledge about the dietary factors associated with multimorbidity has important implications for prevention, diagnosis, treatment and prognosis strategies. The results of this study can contribute to the development of interventions targeting healthy dietary choices when managing multimorbidity.

## Methods

### Study design

Our research analysis is mainly based on the data collected by the United Kingdom Women’s Cohort Study (UKWCS), which has been described in detail in previous literature [22]. The UKWCS initially recruited 35,372 women aged 35–69 years at baseline (1995 to 1998), aiming to explore potential associations between diet and chronic diseases. The dataset in the UKWCS included food consumption, anthropometric measures, socioeconomic status, lifetime lifestyle and health outcomes. Baseline chronic diseases were self-reported by participants including coronary heart disease, angina, stroke, hypertension, hyperlipidemia, diabetes, gallstones, polyps of the large intestine, and any cancers. Considering chronic diseases listed above may make participants more susceptible to multimorbidity during follow-ups, we excluded participants with two or more chronic diseases at baseline in our analyses. Relatively, those living with one chronic disease which were generally not influential were included (*n* = 6,280). The UKWCS has been approved by the National Research Ethics Service (NRES) Committee for Yorkshire & the Humber– Leeds East (Ref: 15/YH/0027) and was updated by the Health Research Authority REC (Reference: 17/YH/0144) for linkage outcomes and related sub-studies.

### Dietary assessment

Baseline dietary data were collected via a self-reported food frequency questionnaire (FFQ) with 217 British food items, which was adapted from the FFQ used in the UK for the European Prospective Investigation into Cancer and Nutrition (EPIC) study [23]. The validation of the FFQ can be seen in previous studies [24]. Food intake frequencies obtained from the FFQ were converted into daily consumed portions for each food item (Supplementary Table 1). Incomplete information on food frequency was assumed that missing items were not consumed. To calculate the weight of each food consumed per day (g/day), daily portions were multiplied by standard portion weights according to the Food Standards Agency portion sizes book [25]. By multiplying the food weight by the standard nutrient composition of foods derived from McCance & Widdowson’s The Composition of Foods (5th Edition) [26], daily energy and nutrient intakes of each food item were calculated and further summed for total energy and nutrients per day consumed for each participant. Nutrient intake from supplements was not accounted for in this study.

The consumption of foods and nutrients was adjusted for total energy using the nutrient density method [27] (for protein, carbohydrates, and fat, the percentage of total energy derived from each one; for other nutrients and foods, the ratio of selected nutrient intake to per MJ of total energy intake). Each energy adjusted intake of nutrients was analyzed in multiple regression models with total energy intake additionally as a covariate using the multivariate nutrient density method recommended by Willett et al. [27].

### Measurement of multimorbidity

We assessed multimorbidity using the CCI, which was proposed by British scholar Charlson in 1987 [28], to assess the impact of multimorbidity on the survival rate of patients in the next 10 years in addition to the underlying diseases. The CCI is the most commonly used tool that quantifies multiple comorbidities. It quantifies the impact of 17 comorbidities based on their numbers and individual prognostic impacts by means of a score [28, 29]. The status of multimorbidity conditions was obtained from the electronic linkages to Hospital Episode Statistics up to March 2019, based on International Classification of Diseases, the Tenth edition, Australian modification (ICD-10-AM) [30], and the CCI was calculated using these data. Diagnostic categories and corresponding ICD-10-AM codes are shown in Supplementary Table 2. In this study, the first primary diagnosis of the disease was excluded from calculation of the CCI as recommended [28, 31, 32]. The second diagnosis or more, which matched the ICD-10-AM codes listed in Supplementary Table 2, was given corresponding scores. The total CCI score was obtained by summing each diagnostic score from the same hospitalization record together. The frequency distribution of the CCI score in participants included in this study is shown in Supplementary Table 3. For the purposes of analysis, CCI scores were categorized as 0 (without multimorbidity), 1 (low level), and greater (moderate to high level). Follow-up of each participant continued until the diagnosis of the main outcome (defined as the occurrence of multimorbidity), death from any cause, or end of the censor date (31 March 2019), whichever came first.

### Covariates

Baseline sociodemographic information, such as age, ethnicity, educational level, and marital status, was obtained by self-report. Body mass index (BMI) was calculated as weight in kilograms divided by height in meters squared. Height and weight were also obtained by self-reporting. Socioeconomic status (SES) was derived from the United Kingdom National Statistics-Socio-Economic Classification (NS-SEC), where participants were classified into three categories (routine/manual, intermediate, or managerial/professional) [33]. Because there were overlapping properties between socioeconomic indicators (education, social class, income, or employment) [34], this study used only SES as an adjustment factor. Participants were asked questions about their daily activities at baseline using the International Physical Activity Questionnaire (IPAQ) short form [35]. According to the official guidelines for data processing and analysis of the IPAQ Short Forms, physical activity was calculated and divided into three levels: low, medium and high. Participants with missing data for any covariates required in the Cox models were excluded, and baseline profiles of nutrient intake were compared between participants with or without missing data in covariates.

### Statistical analysis

To better compare baseline characteristics including sociodemographic, lifestyle, anthropometric and nutritional profiles of the UKWCS participants, each CCI score was categorized into 0 (without multimorbidity), 1 (low level), and greater (moderate to high level) as three CCI groups. The baseline characteristics of the participants are expressed as mean (standard deviation, SD) and number (percentage), and compared among different CCI groups using the ANOVA method and Chi-square tests for continuous and categorical variables, respectively.

Cox’s proportional hazards models were used to estimate associations between daily intakes of nutrients and risk of multimorbidity. The CCI score for each participant was grouped into without (CCI = 0, as reference) or with (CCI > 0) multimorbidity in the COX models. The exposure variables were standardized using a z-score method to make sure comparisons in the same scale, and were further categorized into quintiles of nutrient intake with the lowest quintile (Q1) as the reference. We modeled the median intake of each quintile as a continuous term to test for linear trend. Baseline characteristics were further compared between participants with different intake levels of nutrients that significantly associated with risk of multimorbidity. Adjusted hazard ratios (HR) and 95% confidence intervals (95% CI) in multivariate models for multimorbidity were reported. The covariates included in the multivariate model were age, ethnicity, marital status, SES, physical activity, body mass index, smoking status, alcohol consumption, and total energy intake which were obtained from baseline survey. In this study, the total energy intake was omitted from the covariate set when the exposure factor was energy intake. We conducted subgroup analyses with age 60 as the cutoff point. Interactions terms were tested using the Wald test. In addition, sensitivity analyses were conducted using multinomial logistic regression to test robustness of our results with the CCI = 0 as the base outcome of three CCI groups and each continuous nutrient intake as the exposure. All analyses were carried out using Stata version 17.0, and *p* < 0.05 was considered significant.

## Results

### Socioeconomic characteristics

**Fig. 1:**
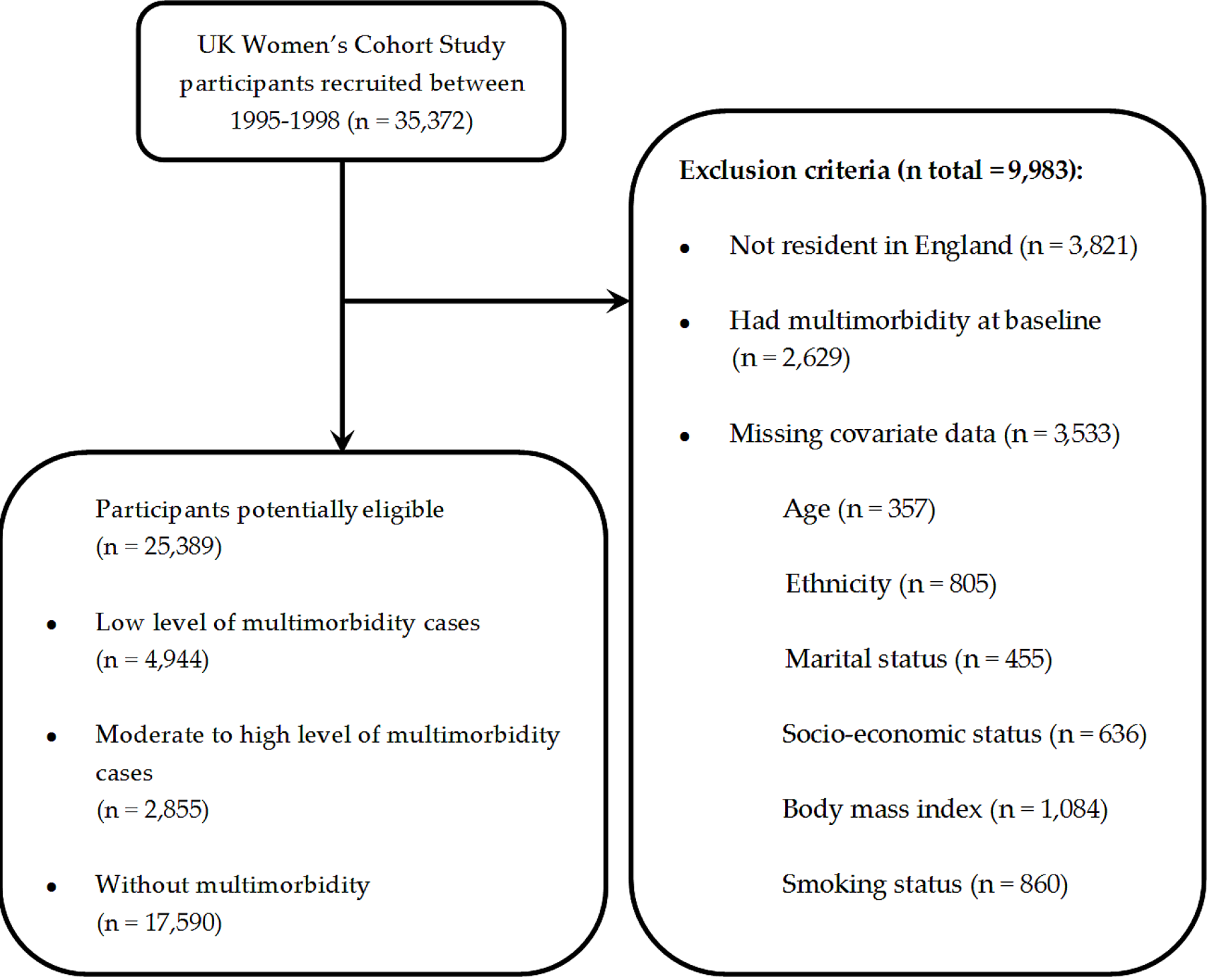
Flow chart of selection of the study population

Of the 35,372 women recruited at recruitment, 3,821 not resident in England, 2,629 living with two or more chronic diseases at baseline, and 3,533 with missing data in covariates were excluded, leaving 25,389 women for analyses in this study (Fig. 1). The mean age of the 25,389 participants was 51 (SD = 9) years. During a mean follow-up of 20 (SD = 5) years (median: 22 years), subjects with a diagnosis of multimorbidity comprised 30.7% of the final study population, including 19.5% with low levels and 11.2% with moderate to high levels. Among the 25,389 participants, 25,080 (98.8%) were White, 131 (0.52%) were Asian, 37 (0.15%) were Black, and 141 (0.60%) were other races. Participants without multimorbidity had a higher educational level and higher levels of SES than their counterparts with multimorbidity (Table 1). Participants with multimorbidity had higher BMI, more ex-smokers and current smokers and were more likely to be single or widowed than those without multimorbidity (Table 1). In addition, although there was a 3-year difference in baseline age, characteristics of most nutrient intake between participants with or without missing covariate data show no significant difference (Supplementary Table 4).

**Table 1:** Demographic characteristics within the UK Women’s Cohort Study*

Characteristics, mean (SD) or n (%)		Charlson Comorbidity Index				Total
		Without multimorbidity (CCI = 0, *N* = 17,590, 69.3%)	Low level (CCI = 1, *N* = 4,944, 19.5%)	Moderate to High level (CCI > 1, *N* = 2,855, 11.2%)	*p*	(*N* = 25,389)
Age at baseline (years)		50 [8]	55 [9]	56 [9]	< 0.001	51 [9]
Follow-up time (years)		22 [2]	14 [6]	14 [6]	< 0.001	20 [5]
Ethnicity (%)	White	17,386 (98.8)	4,875 (98.6)	2,819 (98.7)	0.296	25,080 (98.8)
	Asian	87 (0.50)	32 (0.70)	12 (0.40)		131 (0.52)
	Black	25 (0.10)	10 (0.20)	2 (0.10)		37 (0.15)
	other	92 (0.50)	27 (0.60)	22 (0.80)		141 (0.60)
Educational Level (%)	No qualifications	1,974 (12.0)	1,008 (22.9)	523 (20.6)	< 0.001	3,505 (15.0)
	O-level or equivalent	5,462 (33.0)	1,369 (31.1)	834 (32.8)		7,665 (32.6)
	A-level or equivalent	4,167 (25.2)	1,062 (24.1)	609 (24.0)		5,838 (24.8)
	University degree	4,955 (29.9)	962 (21.9)	576 (22.7)		6,493 (27.6)
Marital status (%)	Married or living as married	13,818 (78.6)	3,647 (73.8)	2,084 (73.0)	< 0.001	19,549 (77.0)
	Separated or divorced	1,888 (10.7)	535 (10.8)	327 (11.5)		2,750 (10.8)
	Single or widowed	1,884 (10.7)	762 (15.4)	444 (15.6)		3,090 (12.2)
Socio-economic status (SES) (%)	Routine and manual	1,429 (8.1)	525 (10.6)	275 (9.6)	< 0.001	2,229 (8.8)
	Intermediate	4,675 (26.6)	1,426 (28.8)	897 (31.4)		6,998 (27.6)
	Professional and managerial	11,486 (65.3)	2,993 (60.5)	1,683 (59.0)		16,162 (63.7)
Physical activity (%)	Low level	1,777 (10.1)	560 (11.3)	309 (10.8)	0.053	2,646 (10.4)
	Moderate level	8,899 (50.6)	2,411 (48.8)	1,411 (49.4)		12,721 (50.1)
	High level	6,914 (39.3)	1,973 (39.9)	1,135 (39.8)		10,022 (39.5)
Body Mass Index (BMI) (kg/m^2^)		24 [4]	25 [5]	25 [4]	< 0.001	24 [4]
Alcohol (g/d)		9 [10]	8 [10]	9 [10]	< 0.001	9 [10]
Smoking status (%)	Never smoked	10,550 (60.0)	2,636 (53.3)	1,584 (55.5)	< 0.001	14,770 (58.2)
	Ex-smoker	5,255 (29.9)	1,666 (33.7)	889 (31.1)		7,810 (30.8)
	Current smoker	1,785 (10.2)	642 (13.0)	382 (13.4)		2,809 (11.1)

* Participants were followed from the baseline survey (1995 to 1998) until the first diagnosis of multimorbidity based on hospital records

### Dietary intake of nutrients

The characteristics of nutrient intake by CCI group are summarized in Table 2. Women without multimorbidity had higher intakes of vitamin B1 and vitamin E but lower intakes of vitamin B2, vitamin B6 and vitamin A than those with different levels of multimorbidity (Table 2). There was little difference in the remaining nutrient intake between the three CCI groups.

**Table 2:** Profiles of nutrient intake within the UK Women’s Cohort Study

Nutrient intake		Charlson Comorbidity Index				Total
		Without multimorbidity (CCI = 0, *N* = 17,590, 69.3%)	Low level (CCI = 1, *N* = 4,944, 19.5%)	Moderate to High level (CCI > 1, *N* = 2,855, 11.3%)	*p*	(*N* = 25,389)
Energy intake	(kcal/day)	2,338 (709)	2,354 (799)	2,375 (1019)	0.041	2,345 (768)
	(MJ/day)	10 [3]	10 [3]	10 [4]	0.041	10 [3]
Protein	(g/day)	88 [28]	91 [34]	91 [39]	< 0.001	89 [31]
	(%energy)	15 [3]	16 [3]	16 [3]	< 0.001	15 [3]
Carbohydrate	(g/day)	310 (103)	312 (112)	314 (133)	0.114	311 (108)
	(%energy)	53 [7]	53 [7]	53 [7]	0.647	53 [7]
Fat	(g/day)	85 [32]	85 [36]	86 [47]	0.034	85 [35]
	(%energy)	32 [6]	32 [6]	32 [6]	0.895	32 [6]
SFAs	(g/day)	29 [13]	30 [14]	30 [17]	< 0.001	29 [14]
	(%energy)	11 [3]	11 [3]	11 [3]	< 0.001	11 [3]
PUFAs	(g/day)	16 [7]	16 [8]	16 [10]	0.077	16 [7]
	(%energy)	6 [2]	6 [2]	6 [2]	< 0.001	6 [2]
MUFAs	(g/day)	28 [11]	28 [12]	28 [16]	0.088	28 [12]
	(%energy)	11 [2]	11 [2]	11 [2]	0.355	11 [2]
Vitamin C	(mg/MJ)	18 [8]	18 [8]	18 [8]	0.513	18 [8]
Vitamin B1	(µg/MJ)	323 (250)	312 (240)	308 (226)	< 0.001	319 (245)
Vitamin B2	(µg/MJ)	262 [66]	269 [69]	269 [68]	< 0.001	264 [67]
Vitamin B6	(µg/MJ)	285 [56]	291 [59]	289 [58]	< 0.001	287 [57]
Vitamin B12	(µg/MJ)	0.58 (0.29)	0.63 (0.31)	0.63 (0.30)	< 0.001	0.59 (0.29)
Folate	(µg/MJ)	42 [9]	42 [10]	42 [10]	< 0.001	42 [10]
Vitamin A	(µg/MJ)	104 [52]	111 [57]	110 [54]	< 0.001	106 [54]
Vitamin D	(µg/MJ)	0.31 (0.15)	0.33 (0.16)	0.33 (0.16)	< 0.001	0.32 (0.15)
Vitamin E	(µg/MJ)	997 (306)	969 (299)	976 (312)	< 0.001	989 (306)
Calcium	(mg/MJ)	119 [28]	121 [29]	119 [29]	0.002	119 [29]
Iron	(mg/MJ)	1.87 (0.55)	1.85 (0.57)	1.88 (0.58)	0.073	1.86 (0.56)
Zinc	(mg/MJ)	1.16 (0.21)	1.19 (0.22)	1.19 (0.22)	< 0.001	1.17 (0.21)

MJ, mega joule; SFAs, saturated fatty acids; PUFAs, polyunsaturated fatty acids; MUFAs, monounsaturated fatty acids

### Associations between nutrient intake and multimorbidity risk

**Fig. 2:**
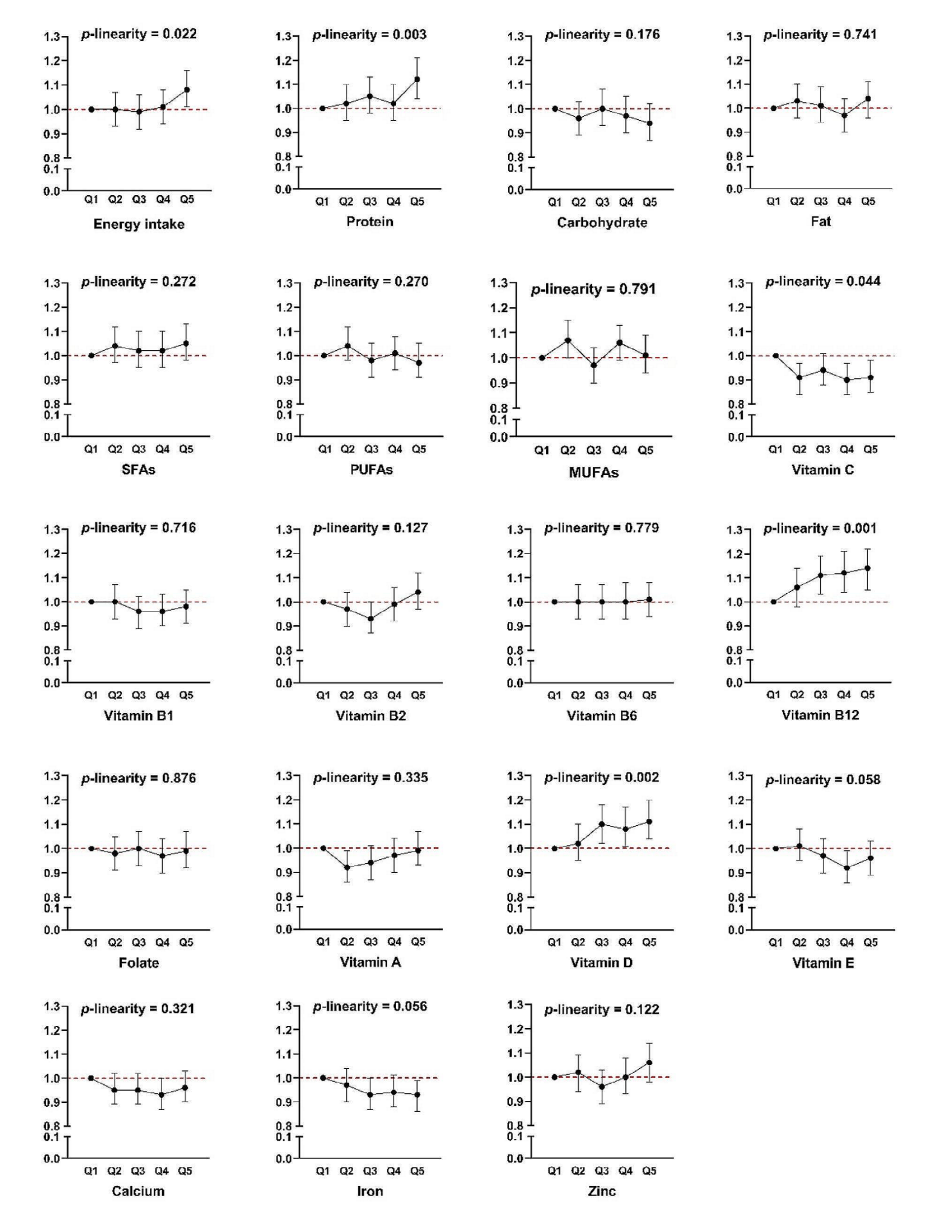
Associations of nutrient intake and risk of multimorbidity within the UK Women’s Cohort Study. The multivariate models adjusted for age, ethnicity, marital status, socioeconomic status, physical activity, body mass index, smoking status, alcohol consumption, and total energy intake. MJ, mega joule; SFAs, saturated fatty acids; PUFAs, polyunsaturated fatty acids; MUFAs, monounsaturated fatty acids. Original data for Fig. 2 were supplemented as Supplementary Table 6 of Appendix 2

The associations between intakes of energy and nutrients and risk of multimorbidity are shown in Fig. 2. Women in the highest quintile of energy and protein intake had 8% and 12% increased risk of multimorbidity respectively compared with women in the lowest quintile (1.08 (1.01, 1.16), *p*-linearity = 0.022 for energy; 1.12 (1.04, 1.21), *p*-linearity = 0.003 for protein). Compared to the lowest quintile, higher quintiles of daily vitamin C intake had around 10% lowered risk of multimorbidity (*p*-linearity = 0.044), while daily vitamin D intake had around 10% increased risk of multimorbidity (*p*-linearity = 0.002).

Compared with the lowest quintile of vitamin B12 intake, the risk of multimorbidity was significantly increased in the highest vitamin B12 quintile (1.14 (1.05, 1.22), *p*-linearity = 0.001). There were some differences in baseline characteristics between participants with different intake levels of nutrients that significantly associated with risk of multimorbidity. For example, compared to participants in the lowest quintile of protein, vitamin B12 and vitamin D intake, those in the highest quintile were more likely to be older, having lower levels of education and SES, having a higher BMI, and less likely to be separated or divorced (Supplementary Table 5). For most other nutrients, no significant associations with multimorbidity risk have been found in this study.

### Subgroup analysis and sensitivity analysis

**Fig. 3:**
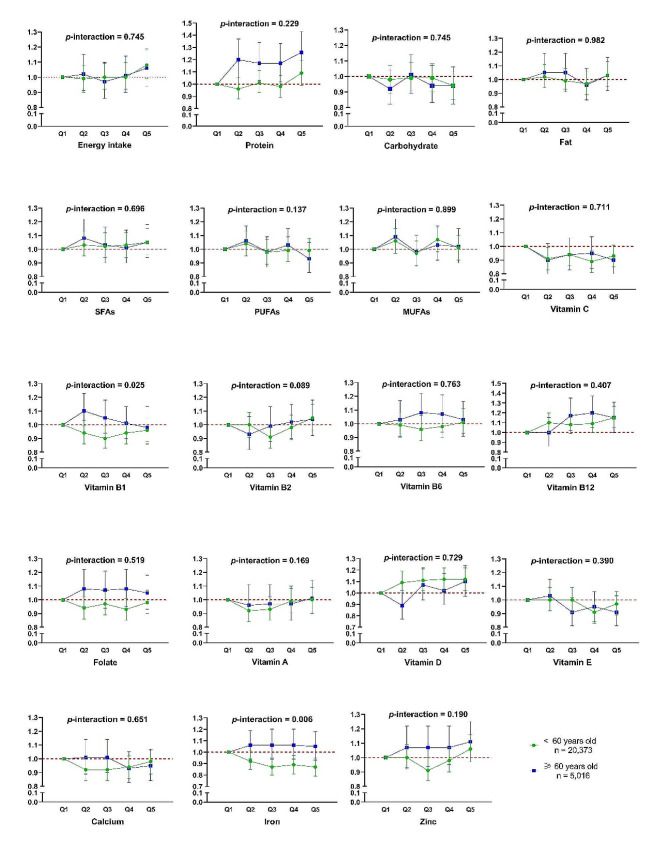
Subgroup analysis by age on associations between nutrient intake and risk of multimorbidity within the UK Women’s Cohort Study. Adjusted for age, ethnicity, marital status, socioeconomic status, physical activity, body mass index, smoking status, alcohol consumption, and total energy intake. *P*-interaction represents the statistical significance for interaction item of dietary factors and age where age was modelled linearly in the Cox proportional regression. 95%CI, 95% Confidence Interval; MJ, mega joule; SFAs, saturated fatty acids; PUFAs, polyunsaturated fatty acids; MUFAs, monounsaturated fatty acids. Original data for Fig. 3 were supplemented as Supplementary Table 8 of Appendix 2

For the subgroup analysis in Fig. 3, there was some evidence of effect modification by age in intakes of vitamin B1 and iron (*p*-interaction = 0.025 and 0.006,) respectively, where there were different trends of association with risk of multimorbidity between participants who were < 60 and ≥ 60 years. For intakes of iron, it was associated with a 11–13% lower risk of multimorbidity in participants aged < 60 years (*p*-interaction = 0.006) only. There was no significant evidence of effect modification by age in intakes of the remaining nutrients (Fig. 3). The results of sensitivity analyses shown in Supplementary Table 7 generally did not contradict our main results. Some nutrients that significantly associated with multimorbidity in COX models became statistically insignificant.

## Discussion

In this study, we found that daily intake of energy and protein was associated with 8% and 12% higher risk of multimorbidity. Daily intake of vitamin C had about 10% lowered risk of multimorbidity, while daily intake of vitamin D had about 10% increased risk of multimorbidity. A significantly higher risk of multimorbidity was found to be associated with the consumption of vitamin B12 in this cohort of UK women. Subgroup analysis showed an effect modification by age in associations between intakes of iron and vitamin B1 and risk of multimorbidity. However, vitamin D and vitamin B12 that significantly associated with multimorbidity in COX models became statistically insignificant in sensitivity analyses, indicating some uncertainty in those associations. We considered it may be caused by the absence of follow-up time periods in multinomial logistic regression analyses, which potentially attenuated the statistical testing power.

Daily intakes of protein increased the multimorbidity risk in our study. Proteins can be absorbed if they are hydrolyzed into amino acids and peptides, which are building blocks of proteins. Recently, studies have indicated that amino acids are potential metabolites that have been associated with aging-related diseases and higher risks of impaired physical functions [36, 37]. Consistent with our report, a population-based cohort study reported that higher concentrations of glutamine and branched-chain amino acids such as isoleucine and valine were associated with higher levels of multimorbidity [38].

At present, the association between iron intake and risk of multimorbidity remains controversial across previous research. Our study showed a negative correlation between iron intake and risk of multimorbidity only in participants aged < 60 years old. A community-based longitudinal study in Malaysia indicated that a one-point increase in daily intakes of iron (mg/day) was associated with decreased chance of developing multimorbidity by 8% among one-disease participants at baseline [39]. In addition, a Chinese study found that daily consumption of iron (mg/day) was lower in people with multimorbidity than in those with one chronic disease or healthy individuals [15]. The result of our subgroup analysis in participants aged < 60 years old is in agreement with these studies. However, high concentrations of labile iron are highly toxic to the cell by generating reactive oxygen species (ROS) that may inflict damage on cells and organs [40]. It was reported that high concentrations of labile iron are highly associated with increased risk of cancer, diabetes, neurodegenerative diseases, liver disease, kidney disease, bone disorders and cardiovascular diseases [41]. Iron seems to accumulate in an age-related and tissue/organ-specific manner, while ferritin levels increase with age [42, 43]. The rate of iron accumulation is proportional to the rate of aging [44]. This might explain why a protective association between iron intake and the risk of multimorbidity was observed in participants aged < 60 years rather than those aged ≥ 60 years in this study.

In our study, we found that higher intakes of vitamin B12 was associated with higher risk of multimorbidity. Although there are few studies that directly looked at associations between vitamin B12 and multimorbidity, the relationship between vitamin B12 and certain diseases plays a role in indirect explanation. Related research has found that high concentrations of vitamin B12 were associated with all-cause mortality [45, 46] and cancer diagnosis/mortality [47]. A prospective longitudinal study named Newcastle 85 + Study from Northeast England found that plasma vitamin B12 in women was associated with an increased risk of mortality in the very elderly [48]. More recently, a study reported that high vitamin B12 intake and high vitamin B12 status were associated with a higher risk of lung cancer [49–51]. Considering that vitamin B12 plays a key role in one-carbon metabolism [52], vitamin B12 is potentially associated with the pathological process of cardiovascular diseases and all-cause mortality. Although potential mechanisms remain unclear, a high level of vitamin B12 in the plasma may result from increased release or decreased clearance of vitamin B12, diminished binding affinity of vitamin B12 for transporter proteins, or upregulation of haptocorrin and transcobalamin synthesis [53, 54].

Recent research has suggested that low levels of vitamin D in plasma are associated with many acute and chronic diseases, such as allergies, depression, cardiovascular disease and cancers [55–57]. A cross-sectional study reported that low plasma vitamin D levels were associated with a higher prevalence of multimorbidity [58]. However, these results differed from ours. The decreasing concentration of 7-dehydrocholesterol in the skin with aging results in a 50% reduction in pre-vitamin D synthesis by older people in response to UV light [59]. Especially in the UK where sunlight time is relatively shorter, sunlight exposure and skin synthesis are probably not enough. Requirements for specific nutrients such as vitamin D are unlikely to be met by the diet alone [60]. Therefore, additional supplements of vitamin D are needed to prevent deficiency. However, our study did not include supplemental intake in the analyses due to incomplete records, which may have contributed to the inconsistent results.

Vitamin C was linked with decreased risk of multimorbidity in this study. Vitamin C is an essential dietary nutrient, which has antioxidant roles and is one of the important antioxidants in human plasma [61, 62]. Kidney failure, peritoneal dialysis, hemodialysis, and various malabsorption disorders are risk factors for a low level of endogenous vitamin C and poor dietary intake of vitamin C in the elderly [63]. Among older individuals with an intake below 75 mg/day, the incidence of various chronic diseases such as diabetes and stroke, was associated with reduced vitamin C intake and plasma status [64, 65]. Although few studies have been reported on associations between vitamin C intake and risk of multimorbidity. Several cross-sectional studies have reported that diets high in fruits and vegetables were associated with a lower risk of multimorbidity [66, 67]. Vegetables and fruits are rich in phytochemicals and nutrients, such as vitamin C and dietary fiber, which may be beneficial to multimorbidity, which potentially supported our results.

### Strengths and limitations

There are several strengths of this study. First, our study used data from a representative sample with a large number of participants and a long follow-up. Second, the linkage to hospital records made it possible to obtain information about most diseases occurred during follow-ups, and minimize loss to follow-up. Third, wide ranges of potential covariates were controlled to provide a better estimate of associations between nutrient intake and risk of multimorbidity.

However, several limitations should also be considered. First, our study did not analyze supplement intake, which may result in biased estimates. Second, our study was restricted to participants of UKWCS that recruited female individuals only, and may therefore not be generalizable to the general population. However, previous studies have shown gender inequality in multimorbidity, where women tend to face a higher risk of multimorbidity than men [68]. Elderly women with lower SES and higher BMI tend to be at higher risk for multimorbidity [68, 69]. Our female cohort is consistent with the fact that women are at high risk for multimorbidity. Third, although many baseline characteristics have been adjusted for as covariates in survival models, there still were some confounders not being fully adjusted for which may result in some bias. Forth, we didn’t exclude participants who were living with one chronic disease at baseline. Participants who have one chronic disease at baseline were more likely to have multimorbidity, which might potentially impact our results. Fifth, this study is mainly made of White ethnic group people (> 98%), which limits our results’ generalizability to other ethnic groups. Therefore, the findings need to be interpreted with caution.

### Implications for policy and practice

Most nutrients are commonly considered beneficial, in particular when they are deficient for individuals. However, our study showed adverse findings for multimorbidity which throws a new aspect of nutrients. The nutrients associated with increased risks of multimorbidity at a higher intake in this study indicated there might be an optimal level of nutrient intakes. The optimal levels of nutrients for individuals with multimorbidity should be further explored, and personalized nutrition should be considered for policy makers and clinical practice. Further clinical trials are needed to determine whether a dietary intervention could exert a beneficial impact on multimorbidity.

## Conclusions

In conclusion, our findings suggest some associations between nutrient intake and risk of multimorbidity. Higher intakes of vitamin B12, vitamin D, protein, and energy may be associated with a high risk of multimorbidity. Conversely, higher intake of vitamin C may be associated with a lower risk of multimorbidity. Daily intake of iron was negatively associated with multimorbidity risk in women aged < 60 years old. There was no evidence of an association between iron intake and multimorbidity in women aged ≥ 60 years in this study. Our findings highlight that some nutrients potentially play a role in risk of multimorbidity, especially vitamin B12, vitamin D, as well as protein and energy.

### Electronic supplementary material

Below is the link to the electronic supplementary material.

Supplementary Material 1

Supplementary Material 2

## Data Availability

The data that support the findings of this study are available from Leeds Analytic Secure Environment for Research but restrictions apply to the availability of these data, which were used under license for the current study, and so are not publicly available. Data are however available from the corresponding author upon reasonable request and with permission of Leeds Analytic Secure Environment for Research.

## Abbreviations

CCI: Charlson comorbidity index
UKWCS: United Kingdom Women’s Cohort Study
NRES: National Research Ethics Service
FFQ: food frequency questionnaire
EPIC: European Prospective Investigation into Cancer and Nutrition
ICD-10-AM: Tenth edition, Australian modification
SD: standard deviation
HR: hazard ratios
95% CI: 95% confidence intervals
SES: socioeconomic status
BMI: body mass index
NS-SEC: National Statistics-Socio-Economic Classification
IPAQ: International Physical Activity Questionnaire
MJ: mega joule
SFAs: saturated fatty acids
PUFAs: polyunsaturated fatty acids
MUFAs: monounsaturated fatty
ROS: reactive oxygen species
CVD: cardiovascular disease

## References

[1] Johnston MC, Crilly M, Black C, Prescott GJ, Mercer SW (2019). Defining and measuring multimorbidity: a systematic review of systematic reviews. Eur J Public Health.

[2] Moffat K, Mercer SW (2015). Challenges of managing people with multimorbidity in today’s healthcare systems. Bmc Fam Pract.

[3] Marengoni A, Angleman S, Melis R, Mangialasche F, Karp A, Garmen A (2011). Aging with multimorbidity: a systematic review of the literature. Ageing Res Rev.

[4] King DE, Xiang J, Pilkerton CS (2018). Multimorbidity trends in United States adults, 1988–2014. J Am Board Fam Med.

[5] Kingston A, Robinson L, Booth H, Knapp M, Jagger C, project M (2018). Projections of multi-morbidity in the older population in England to 2035: estimates from the Population Ageing and Care Simulation (PACSim) model. Age Ageing.

[6] Wang HH, Wang JJ, Wong SY, Wong MC, Li FJ, Wang PX (2014). Epidemiology of multimorbidity in China and implications for the healthcare system: cross-sectional survey among 162,464 community household residents in southern China. BMC Med.

[7] Smith SM, Soubhi H, Fortin M, Hudon C, O’Dowd T (2012). Managing patients with multimorbidity: systematic review of interventions in primary care and community settings. Bmj-Brit Med J.

[8] Nunes BP, Flores TR, Mielke GI, Thume E, Facchini LA (2016). Multimorbidity and mortality in older adults: a systematic review and meta-analysis. Arch Gerontol Geriatr.

[9] Bowling CB, Deng L, Sakhuja S, Morey MC, Jaeger BC, Muntner P (2019). Prevalence of Activity Limitations and Association with Multimorbidity among US Adults 50 to 64 Years Old. J Gen Intern Med.

[10] Pathirana TI, Jackson CA (2018). Socioeconomic status and multimorbidity: a systematic review and meta-analysis. Aust Nz J Publ Heal.

[11] Dugravot A, Fayosse A, Dumurgier J, Bouillon K, Ben Rayana T, Schnitzler A (2020). Social inequalities in multimorbidity, frailty, disability, and transitions to mortality: a 24-year follow-up of the Whitehall II cohort study. Lancet Public Health.

[12] Palmer K, Marengoni A, Forjaz MJ, Jureviciene E, Laatikainen T, Mammarella F (2018). Multimorbidity care model: recommendations from the consensus meeting of the Joint Action on Chronic diseases and promoting healthy ageing across the life cycle (JA-CHRODIS). Health Policy.

[13] Zheng Y, Zhou Z, Wu T, Zhong K, Hu H, Zhang H (2023). Association between composite lifestyle factors and cardiometabolic multimorbidity in Chongqing, China: a cross-sectional exploratory study in people over 45 years and older. Front Public Health.

[14] Dekker LH, de Borst MH, Meems LMG, de Boer RA, Bakker SJL, Navis GJ (2019). The association of multimorbidity within cardio-metabolic disease domains with dietary patterns: a cross-sectional study in 129 369 men and women from the Lifelines cohort. PLoS ONE.

[15] Ruel G, Shi ZM, Zhen SQ, Zuo H, Kroger E, Sirois C (2014). Association between nutrition and the evolution of multimorbidity: the importance of fruits and vegetables and whole grain products. Clin Nutr.

[16] Freisling H, Viallon V, Lennon H, Bagnardi V, Ricci C, Butterworth AS (2020). Lifestyle factors and risk of multimorbidity of cancer and cardiometabolic diseases: a multinational cohort study. BMC Med.

[17] Kyprianidou M, Panagiotakos D, Faka A, Kambanaros M, Makris KC, Christophi CA (2021). Adherence to the Mediterranean diet in Cyprus and its relationship to multi-morbidity: an epidemiological study. Public Health Nutr.

[18] Vicinanza R, Bersani FS, D’Ottavio E, Murphy M, Bernardini S, Crisciotti F (2020). Adherence to Mediterranean diet moderates the association between multimorbidity and depressive symptoms in older adults. Arch Gerontol Geriat.

[19] Zhang Y, Chen H, Carrillo-Larco RM, Lim CCW, Mishra SR, Yuan CZ (2022). Association of dietary patterns and food groups intake with multimorbidity: a prospective cohort study. Clin Nutr Espen.

[20] Jeong D, Kim J, Lee H, Kim D, Lim H. Association of Cardiometabolic Multimorbidity Pattern with Dietary factors among adults in South Korea. Nutrients. 2020;12(9). 10.3390/nu12092730 PMC7551044 32906713

[21] Zhao HT, Wang CC, Pan YG, Guo YP, Yao N, Wang H et al. Niacin, lutein and zeaxanthin and physical activity have an impact on Charlson comorbidity index using zero-inflated negative binomial regression model: National Health and Nutrition Examination Survey 2013–2014. BMC Public Health. 2019;19(1). 10.1186/s12889-019-7906-7 PMC6883694 31779602

[22] Cade JE, Burley VJ, Alwan NA, Hutchinson J, Hancock N, Morris MA (2017). Cohort Profile: the UK women’s Cohort Study (UKWCS). Int J Epidemiol.

[23] Riboli E, Kaaks R (1997). The EPIC project: Rationale and study design. European prospective investigation into Cancer and Nutrition. Int J Epidemiol.

[24] Cade JE, Burley VJ, Greenwood DC, G UWsCSS (2004). The UK women’s Cohort Study: comparison of vegetarians, fish-eaters and meat-eaters. Public Health Nutr.

[25] Food Standards Agency (FSA) (2002). Food Portion sizes.

[26] Holland B, Welch AA, Unwin ID, Buss DH, Paul AA, Southgate D (1991). McCance and Widdowson’s the composition of Foods.

[27] Willett W. Nutritional epidemiology. 3rd ed. Oxford University Press; 2012.

[28] Charlson ME, Pompei P, Ales KL, MacKenzie CR (1987). A new method of classifying prognostic comorbidity in longitudinal studies: development and validation. J Chronic Dis.

[29] Charlson M, Szatrowski TP, Peterson J, Gold J (1994). Validation of a combined comorbidity index. J Clin Epidemiol.

[30] Sundararajan V, Henderson T, Perry C, Muggivan A, Quan H, Ghali WA (2004). New ICD-10 version of the Charlson comorbidity index predicted in-hospital mortality. J Clin Epidemiol.

[31] Yurkovich M, Avina-Zubieta JA, Thomas J, Gorenchtein M, Lacaille D (2015). A systematic review identifies valid comorbidity indices derived from administrative health data. J Clin Epidemiol.

[32] Prasad B, Bjourson AJ, Shukla P. Data-driven patient stratification of UK Biobank cohort suggests five endotypes of multimorbidity. Brief Bioinform. 2022;23(6). 10.1093/bib/bbac410 PMC9677496 36209412

[33] The National Statistics Socio-Economic Classification User Manual Office for National Statistics. https://www.ons.gov.uk/ons/guide-method/classifications/archived-standard-classifications/soc-and-sec-archive/the-national-statistics-socio-economic-classification--user-manual.pdf (Accessed on 30 Oct 2023).

[34] Darin-Mattsson A, Fors S, Kareholt I (2017). Different indicators of socioeconomic status and their relative importance as determinants of health in old age. Int J Equity Health.

[35] Eveleth PB (1996). Physical status: the use and interpretation of anthropometry. Report of a WHO Expert Committee - WHO. Am J Hum Biol.

[36] Timmerman KL, Volpi E (2008). Amino acid metabolism and regulatory effects in aging. Curr Opin Clin Nutr.

[37] Caballero FF, Struijk EA, Buno A, Vega-Cabello V, Rodriguez-Artalejo F, Lopez-Garcia E (2022). Plasma amino acids and risk of impaired lower-extremity function and role of Dietary Intake: a nested case-control study in older adults. Gerontology.

[38] Caballero FF, Lana A, Struijk EA, Arias-Fernandez L, Yevenes-Briones H, Cardenas-Valladolid J (2023). Prospective Association between plasma amino acids and multimorbidity in older adults. J Gerontol a-Biol.

[39] Hussin NM, Shahar S, Din NC, Singh DKA, Chin AV, Razali R (2019). Incidence and predictors of multimorbidity among a multiethnic population in Malaysia: a community-based longitudinal study. Aging Clin Exp Res.

[40] Cabantchik ZI, Breuer W, Zanninelli G, Cianciulli R (2005). LPI-labile plasma iron in iron overload. Best Pract Res Cl Ha.

[41] Dev S, Babitt JL (2017). Overview of iron metabolism in health and disease. Hemodial Int.

[42] Wang CY, Jenkitkasemwong S, Duarte S, Sparkman BK, Shawki A, Mackenzie B (2012). ZIP8 is an iron and zinc transporter whose cell-surface expression is up-regulated by cellular iron loading. J Biol Chem.

[43] Wang D, Li WB, Wei XE, Li YH, Dai YM (2012). An investigation of age-related iron deposition using susceptibility weighted imaging. PLoS ONE.

[44] Massie HR, Aiello VR, Williams TR (1985). Iron Accumulation during Development and Aging of Drosophila. Mech Ageing Dev.

[45] Salles N, Herrmann F, Sieber C, Rapin C (2008). High vitamin B12 level and mortality in elderly inpatients. J Nutr Health Aging.

[46] Gonzalez S, Huerta JM, Fernandez S, Patterson AM, Lasheras C (2007). Homocysteine increases the risk of mortality in elderly individuals. Brit J Nutr.

[47] Arendt JFB, Pedersen L, Nexo E, Sorensen HT (2013). Elevated plasma vitamin B12 levels as a marker for Cancer: a Population-based Cohort Study. Jnci-J Natl Cancer I.

[48] Mendonca N, Jagger C, Granic A, Martin-Ruiz C, Mathers JC, Seal CJ (2018). Elevated total homocysteine in all participants and plasma vitamin B12 concentrations in women are Associated with all-cause and Cardiovascular Mortality in the very old: the Newcastle 85 + study. J Gerontol a-Biol.

[49] Ebbing M, Bonaa KH, Nygard O, Arnesen E, Ueland PM, Nordrehaug JE (2009). Cancer incidence and mortality after treatment with folic acid and vitamin B12. JAMA.

[50] Brasky TM, White E, Chen CL, Long-Term S (2017). Metabolism-related vitamin B use in relation to Lung Cancer Risk in the vitamins and Lifestyle (VITAL) cohort. J Clin Oncol.

[51] Fanidi A, Carreras-Torres R, Larose TL, Yuan JM, Stevens VL, Weinstein SJ (2019). Is high vitamin B12 status a cause of lung cancer?. Int J Cancer.

[52] McGarel C, Pentieva K, Strain JJ, McNulty H (2015). Emerging roles for folate and related B-vitamins in brain health across the lifecycle. Proc Nutr Soc.

[53] Andres E, Serraj K, Zhu J, Vermorken AJ (2013). The pathophysiology of elevated vitamin B12 in clinical practice. QJM.

[54] Quadros EV, Sequeira JM (2013). Cellular uptake of cobalamin: transcobalamin and the TCblR/CD320 receptor. Biochimie.

[55] Hossein-nezhad A, Holick MF (2013). Vitamin D for Health: A Global Perspective. Mayo Clin Proc.

[56] Holick MF (2007). Vitamin D deficiency. New Engl J Med.

[57] Giovannucci E (2013). Epidemiology of vitamin D and colorectal cancer. Anti-Cancer Agent Me.

[58] Meems LM, de Borst MH, Postma DS, Vonk JM, Kremer HP, Schuttelaar ML (2015). Low levels of vitamin D are associated with multimorbidity: results from the LifeLines Cohort Study. Ann Med.

[59] Kinyamu HK, Gallagher JC, Petranick KM, Ryschon KL (1996). Effect of parathyroid hormone (hPTH[1–34]) infusion on serum 1,25-dihydroxyvitamin D and parathyroid hormone in normal women. J Bone Min Res.

[60] Saternus R, Vogt T, Reichrath J. A critical Appraisal of strategies to optimize vitamin D status in Germany, a Population with a western Diet. Nutrients. 2019;11(11). 10.3390/nu11112682 PMC6893762 31698703

[61] Padayatty SJ, Katz A, Wang YH, Eck P, Kwon O, Lee JH (2003). Vitamin C as an antioxidant: evaluation of its role in disease prevention. J Am Coll Nutr.

[62] Halliwell B (1996). Vitamin C: antioxidant or pro-oxidant in vivo?. Free Radic Res.

[63] Hemilä H, Chalker E. Vitamin C may reduce the duration of mechanical ventilation in critically ill patients: a meta-regression analysis. J Intensive Care. 2020;8(1). 10.1186/s40560-020-0432-y PMC7006137 32047636

[64] Lykkesfeldt J. On the effect of vitamin C intake on human health: how to (mis)interprete the clinical evidence. Redox Biol. 2020;34. 10.1016/j.redox.2020.101532 PMC7296342 32535545

[65] Sun HB, Karp J, Sun KM, Weaver CM, Decreasing Vitamin C, Intake (2022). Low serum vitamin C level and risk for US adults with diabetes. Nutrients.

[66] Wikström K, Lindström J, Harald K, Peltonen M, Laatikainen T (2015). Clinical and lifestyle-related risk factors for incident multimorbidity: 10-year follow-up of Finnish population-based cohorts 1982–2012. Eur J Intern Med.

[67] Ruel G, Shi ZM, Zhen SQ, Zuo H, Kröger E, Sirois C (2014). Association between nutrition and the evolution of multimorbidity: the importance of fruits and vegetables and whole grain products. Clin Nutr.

[68] Violan C, Foguet-Boreu Q, Flores-Mateo G, Salisbury C, Blom J, Freitag M (2014). Prevalence, determinants and patterns of multimorbidity in primary care: a systematic review of observational studies. PLoS ONE.

[69] Blumel JE, Carrillo-Larco RM, Vallejo MS, Chedraui P (2020). Multimorbidity in a cohort of middle-aged women: risk factors and disease clustering. Maturitas.

