# Supplementary material for: Nutrient intake and risk of multimorbidity: a prospective cohort study of 25,389 women
Source: BMC Public Health. 2024 Mar 4;24:696. doi: 10.1186/s12889-024-18191-9 (PMC10913224; doi:10.1186/s12889-024-18191-9)
Supplement: Supplementary file 1 — Supplementary Material 1 [file 12889_2024_18191_MOESM1_ESM.docx]

**Appendix 1**

Contents

**Supplementary tables**2-10

Supplementary Table 1. Transformation from intake frequencies to portion servings. 2

Supplementary Table 2. Diagnostic categories, original ICD-9-CM codes, and corresponding ICD-10-AM codes. 3-4

Supplementary Table 3. The frequency distribution of the CCI score in UKWCS participants included in this study.5

Supplementary Table 4. Baseline profiles of nutrient intake between participants with or without missing data in covariates.6

Supplementary Table 5. Demographic characteristics of UKWCS participants with different quintiles of protein, vitamin B12 and vitamin D intake.7-9

Supplementary Table 7. Associations between nutrient intake and risk of multimorbidity with different levels using multinomial logistic regression analysis.10

Supplementary Table 1. Transformation from intake frequencies to portion servings. per day

| **Frequency of intake** | **Portion servings per day (formula)** |
| --- | --- |
| Never | 0 |
| Less than once a month | 0.02 (0.5/30) |
| 1-3 per month | 0.07 (2.0/30) |
| Once a week | 0.14 (1.0/7) |
| 2-4 per week | 0.40 (3.0/7) |
| 5-6 per week | 0.80 (5.5/7) |
| Once per day | 1.00 (1.0/1) |
| 2-3 per day | 2.50 (2.5/1) |
| 4-5 per day | 4.50 (4.5/1) |
| 6+ per day | 6.00 (6.0/1) |

Supplementary Table 2. Diagnostic categories, original ICD-9-CM codes, and corresponding ICD-10-AM codes*.

| **Codes** | | | |
| --- | --- | --- | --- |
| **Condition** | **Weights** | **ICD-9-CM** | **ICD-10-AM** |
| Acute myocardial infarction | 1 | 410, 412 | I21, I22, I252 |
| Congestive heart failure | 1 | 428 | I50 |
| Peripheral vascular disease | 1 | 441, 4439, 7854, V434 | I71, I790, I739, R02, Z958, Z959 |
| Cerebral vascular accident | 1 | 430–438 | I60, I61, I62, I63, I65, I66,G450, G451, G452, G458, G459, G46, I64, G454, I670, I671, I672, I674, I675, I676, I677 I678, I679, I681, I682, I688, I69 |
| Dementia | 1 | 290 | F00, F01, F02, F051 |
| Pulmonary disease | 1 | 490, 491, 492, 493, 494, 495, 496, 500, 501, 502, 503, 504, 505 | J40, J41, J42, J44, J43, J45, J46, J47, J67, J44, J60, J61, J62, J63, J66, J64, J65 |
| Connective tissue disorder | 1 | 7100, 7101, 7104, 7140, 7141, 7142, 71481(now5171), 725 | M32,M34,M332,M053,M058, M059,M060,M063, M069, M050, M052, M051, M353 |
| Peptic ulcer | 1 | 531, 532, 533, 534 | K25, K26, K27, K28 |
| Liver disease | 1 | 5712, 5714, 5715, 5716 | K702, K703, K73, K717, K740, K742, K746, K743, K744, K745 |
| Diabetes | 1 | 2500, 2501, 2502, 2503, 2507 | E109, E119, E139, E149, E101, E111, E131, E141, E105, E115, E135, E145 |
| Diabetes complications | 2 | 2504, 2505, 2506 | E102, E112, E132, E142 E103, E113, E133, E143 E104, E114, E134, E144 |
| Paraplegia | 2 | 342, 3441 | 342, 3441 |
| Renal disease |  | 582, 5830, 5831, 5832, 5833, 5835, 5836, 5837, 5834, 5855, 5865 | N03, N052, N053, N054, N055, N056, N072, N073, N074, N01, N18, N19, N25 |
| Cancer | 2 | 14, 15, 16, 18, 170, 171, 172, 174, 175, 176, 179, 190, 191, 192, 193, 194, 1950, 1951, 1952, 1953, 1954, 1955, 1958, 200, 201, 202, 203, 204, 205, 206, 207, 208 | C0, C1, C2, C3, C40, C41, C43, C45, C46, C47, C48, C49, C5, C6, C70, C71, C72, C73, C74, C75, C76, C80, C81, C82, C83, C84, C85, C883, C887, C889, C900, C901, C91, C92, C93, C940, C941, C942, C943, C9451, C947, C95, C96 |
| Metastatic cancer | 3 | 196, 197, 198, 1990, 1991 | C77, C78, C79, C80 |
| Severe liver disease | 3 | 5722, 5723, 5724, 5728 | K729, K766, K767, K721 |
| HIV | 6 | 042, 043, 044 | B20, B21, B22, B23, B24 |

ICD-9-CM, International Classification of Diseases, Ninth Revision, Clinical Modification; ICD-10-AM, International Classification of Diseases, Tenth edition, Australian modification; HIV, human immunodeficiency virus.

* Sundararajan V, Henderson T, Perry C, Muggivan A, Quan H, Ghali WA. New ICD-10 version of the Charlson comorbidity index predicted in-hospital mortality. J Clin Epidemiol. 2004;57(12):1288-94.

Supplementary Table 3. The frequency distribution of the CCI score in UKWCS participants included in this study.

| **CCI score** | **Number** | **Percentage** | **Cumulative Percentage** |
| --- | --- | --- | --- |
| 0 | 17,590 | 69.28 | 69.28 |
| 1 | 4,944 | 19.47 | 88.75 |
| 2 | 1,509 | 5.94 | 94.70 |
| 3 | 928 | 3.66 | 98.35 |
| 4 | 96 | 0.38 | 98.73 |
| 5 | 95 | 0.37 | 99.11 |
| 6 | 131 | 0.52 | 99.62 |
| 7 | 11 | 0.04 | 99.67 |
| 8 | 20 | 0.08 | 99.74 |
| 9 | 33 | 0.13 | 99.87 |
| 10 | 6 | 0.02 | 99.90 |
| 11 | 6 | 0.02 | 99.92 |
| 12 | 7 | 0.03 | 99.95 |
| 13 | 3 | 0.01 | 99.96 |
| 14 | 2 | 0.01 | 99.97 |
| 15 | 5 | 0.02 | 99.99 |
| 16 | 0 | 0.00 | 99.99 |
| 17 | 0 | 0.00 | 99.99 |
| 18 | 2 | 0.01 | 100.00 |
| 19 | 1 | 0.00 | 100.00 |
| Total | 25,389 | 100.00 |  |

| **Characteristics, mean (SD) or n (%)** | | **25,389 potentially eligible participants** | **3,533 participants excluded from adjusted analyses** | ***P*** |
| --- | --- | --- | --- | --- |
| Age at baseline (years) | | 51 (9) | 54 (10) | <0.001 |
| Follow-up time (years) | | 20 (5) | 19 (6) | <0.001 |
| Body Mass Index (BMI) (kg/m^2^) | | 24 (4) | 24 (4) | 0.918 |
| Energy intake  Intake | (kcal/day) | 2345 (768) | 2305 (840) | <0.001 |
|  | (MJ/day) | 10 (3) | 10 (4) | <0.001 |
| Protein | (g/day) | 89 (31) | 87 (32) | <0.001 |
|  | (%energy) | 15 (3) | 15 (3) | 0.061 |
| Carbohydrate | (g/day) | 311 (108) | 309 (121) | 0.236 |
|  | (%energy) | 53 (7) | 54 (7) | <0.001 |
| Fat | (g/day) | 85 (35) | 83 (38) | 0.019 |
|  | (%energy) | 32 (6) | 32 (6) | 0.762 |
| SFAs | (g/day) | 29 (14) | 29 (15) | 0.004 |
|  | (%energy) | 11 (3) | 11 (3) | 0.194 |
| PUFAs | (g/day) | 16 (7) | 16 (8) | 0.029 |
|  | (%energy) | 6 (2) | 6 (2) | 0.611 |
| MUFAs | (g/day) | 28 (12) | 27 (13) | 0.017 |
|  | (%energy) | 11 (2) | 11 (2) | 0.487 |
| Vitamin C (mg/MJ) | | 18 (8) | 18 (9) | <0.001 |
| Vitamin B1 (mg/MJ) | | 319 (245) | 321 (271) | 0.744 |
| Vitamin B2 (μg/MJ) | | 264 (67) | 263 (74) | 0.284 |
| Vitamin B6 (μg/MJ) | | 287 (57) | 288 (63) | 0.084 |
| Vitamin B12 (μg/MJ) | | 0.59 (0.29) | 0.58 (0.31) | 0.023 |
| Folate (μg/MJ) | | 42 (10) | 42 (11) | 0.003 |
| Vitamin A (μg/MJ) | | 106 (54) | 108 (58) | 0.045 |
| Vitamin D (μg/MJ) | | 0.32 (0.15) | 0.32 (0.17) | 0.100 |
| Vitamin E (μg/MJ) | | 989 (306) | 994 (318) | 0.369 |
| Calcium (mg/MJ) | | 119 (29) | 118 (31) | 0.006 |
| Iron (mg/MJ) | | 1.86 (0.56) | 1.87 (0.60) | 0.599 |
| Zinc (mg/MJ) | | 1.17 (0.21) | 1.17 (0.22) | 0.512 |

Supplementary Table 4. Baseline profiles of nutrient intake between participants with or without missing data in covariates.

MJ, mega joule; SFAs, saturated fatty acids; PUFAs, polyunsaturated fatty acids; MUFAs, monounsaturated fatty acids

Supplementary Table 5. Demographic characteristics of UKWCS participants with different quintiles of protein, vitamin B12 and vitamin D intake.

| **Characteristics, mean (SD) or**  **n (%)** | | **Nutrient intake** | | | | | | | | | | | | | | | | | | |  |
| --- | --- | --- | --- | --- | --- | --- | --- | --- | --- | --- | --- | --- | --- | --- | --- | --- | --- | --- | --- | --- | --- |
|  |  | **Protein** | | | | | | | **Vitamin B12** | | | | | | **Vitamin D** | | | | | |  |
|  |  | **Q1** | **Q2** | **Q3** | **Q4** | **Q5** | | ***p*** | **Q1** | **Q2** | **Q3** | **Q4** | **Q5** | ***p*** | **Q1** | **Q2** | **Q3** | **Q4** | **Q5** | ***p*** | |
| Age at baseline (years) | | 49 (9) | 50 (9) | 52 (9) | 52 (9) | | 53 (9) | <0.001 | 49 (8) | 50 (9) | 52 (9) | 53 (9) | 54 (9) | <0.001 | 49 (8) | 51 (9) | 52 (9) | 52 (9) | 53 (9) | <0.001 |  |
| Follow-up time (years) | | 20 (5) | 20 (5) | 20 (5) | 19 (5) | | 19 (6) | <0.001 | 21 (5) | 20 (5) | 20 (5) | 19 (5) | 19 (6) | <0.001 | 20 (5) | 20 (5) | 19 (5) | 19 (5) | 19 (6) | <0.001 |  |
| Ethnicity (%) | White | 4,975  (97.97) | 5,000  (98.46) | 5,031  (99.07) | 5,039  (99.23) | | 5,035  (99.17) | <0.001 | 4,940  (97.28) | 5,008  (98.62) | 5,037  (99.19) | 5,046  (99.37) | 5 ,049  (99.45) | <0.001 | 4,949  (97.46) | 5,022  (98.90) | 5,031  (99.07) | 5,034  (99.13) | 5,044  (99.35) | <0.001 |  |
|  | Asian | 59  （1.16） | 36  (0.71) | 12  (0.24) | 14  (0.28) | | 10  (0.20) |  | 79  (1.56) | 27  (0.53) | 11  (0.22) | 8  (0.16) | 6  (0.12) |  | 80  (1.58) | 18  (0.35) | 14  (0.28) | 10  (0.20) | 9  (0.18) |  |  |
|  | Black | 6  (0.12) | 9 (0.18) | 9  (0.18) | 6  (0.12) | | 7  (0.14) |  | 11  (0.22) | 9  (0.18) | 8  (0.16) | 3  (0.06) | 6  (0.32) |  | 8  (0.16) | 5  (0.10) | 6  (0.12) | 11  (0.22) | 7  (0.14) |  |  |
|  | other | 38  (0.75) | 33  (0.65) | 26  (0.51) | 19  (0.37) | | 25  (0.49) |  | 48  (0.95) | 34  (0.67) | 22  (0.43) | 21  (0.41) | 16  (0.32) |  | 41  (0.81) | 33  (0.65) | 27  (0.53) | 23  (0.45) | 17  (0.33) |  |  |
| Educational Level (%) | No  qualifications | 517  (10.80) | 565  (11.87) | 691  (14.74) | 769  (16.50) | | 963  (20.93) | <0.001 | 502  (10.46) | 638  (13.49) | 725  (15.39) | 736  (15.86) | 904  (19.58) | <0.001 | 512  (10.68) | 653  (13.75) | 666  (14.20) | 791  (16.99) | 883  (19.13) | <0.001 |  |
|  | O-level or equivalent | 1,224  (25.56) | 1,462  (30.71) | 1,559  (33.25) | 1,693  (36.32) | | 1,727  (37.53) |  | 1,294  (25.21) | 1,475  (31.18) | 1,591  (33.76) | 1,688  (36.37) | 1,617  (35.02) |  | 1,375  (28.68) | 1,516  (31.93) | 1,580  (33.70) | 1,603  (34.44) | 1,591  (34.47) |  |  |
|  | A-level or equivalent | 1,237  (25.84) | 1,220  (25.62) | 1,164  (24.82) | 1,139  (24.44) | | 1,078  (23.42) |  | 1,210  (25.21) | 1,156  (24.43) | 1,193  (25.32) | 1,144  (24.65) | 1,135  (24.58) |  | 1,178  (24.57) | 1,157  (24.37) | 1,252  (26.70) | 1,167  (25.07) | 1,084  (23.49) |  |  |
|  | University degree | 1,810  (37.80) | 1,514  (31.80) | 1,275  (27.19) | 1,060  (22.74) | | 834  (18.12) |  | 1,794  (37.38) | 1,462  (30.90) | 1,203  (25.53) | 1,073  (23.12) | 961  (20.81) |  | 1,729  (36.07) | 1,422  (29.95) | 1,191  (25.40) | 1,094  (23.50) | 1,057  (22.90) |  |  |
| Marital status (%) | Married or living as married | 3,597  (70.83) | 3,815  (75.13) | 4,015  (79.07) | 3,982  (78.42) | | 4,140  (81.54) | <0.001 | 3,596  (70.82) | 3,899  (76.78) | 4,008  (78.93) | 4,039  (79.54) | 4,007  (78.92) | <0.001 | 3,644  (71.76) | 3,907  (76.94) | 4,016  (79.09) | 4,031  (79.38) | 3,951  (77.82) | <0.001 |  |
|  | Separated or divorced | 723  (14.24) | 646  (12.72) | 513  (10.10) | 486  (9.57) | | 382  (7.52) |  | 772  (15.20) | 605  (11.91) | 475  (9.35) | 458  (9.02) | 440  (8.67) |  | 772  (15.20) | 593  (11.68) | 475  (9.35) | 458  (9.02) | 452  (8.90) |  |  |
|  | Single or widowed | 758  (14.93) | 617  (12.15) | 550  (10.83) | 610  (12.01) | | 555  (10.93) |  | 710  (13.98) | 574  (11.30) | 595  (11.72) | 581  (11.44) | 630  (12.41) |  | 662  (13.04) | 578  (11.38) | 587  (11.56) | 589  (11.60) | 674  (13.28) |  |  |
| Socio-economic status (SES) (%) | Routine and manual | 411  (8.09) | 437  (8.61) | 426  (8.39) | 459  (9.04) | | 496  (9.77) | <0.001 | 404  (7.96) | 439  (8.65) | 457  (9.00) | 463  (9.12) | 466  (9.18) | <0.001 | 352  (6.93) | 432  (8.51) | 475  (9.35) | 460  (9.06) | 540  (10.05) | <0.001 |  |
|  | Intermediate | 1,133  (22.31) | 1,304  (25.68) | 1,404  (27.65) | 1,524  (30.01) | | 1,633  (32.16) |  | 1,120  (22.06) | 1,312  (25.84) | 1,498  (29.5) | 1,486  (29.26) | 1,582  (31.16) |  | 1,164  (22.92) | 1,321  (26.01) | 1,411  (27.79) | 1,569  (30.90) | 1,533  (30.19) |  |  |
|  | Professional and managerial | 3,534  (69.59) | 3,337  (65.71) | 3,248  (63.96) | 3,095  (60.95) | | 2,948  (58.07) |  | 3,554  (69.99) | 3,327  (65.52) | 3,123  (61.50) | 3,129  (61.62) | 3,029  (59.66) |  | 3,562  (70.15) | 3,325  (65.48) | 3,192  (62.86) | 3,049  (60.04) | 3,034  (59.76) |  |  |
| Physical  activity  (%) | Low level | 512  (10.08) | 494  (9.73) | 521  (10.26) | 538  (10.59) | | 581  (11.44) | 0.028 | 487  (9.59) | 564  (11.11) | 538  (10.59) | 537  (10.58) | 520  (10.24) | <0.001 | 510  (10.04) | 537  (10.58) | 518  (10.20) | 548  (10.79) | 533  (10.50) | <0.001 |  |
|  | Moderate level | 2,471  (48.66) | 2,570  (50.61) | 2,573  (50.67) | 2,561  (50.43) | | 2,546  (50.15) |  | 2,422  (47.70) | 2,566  (50.53) | 2,639  (51.97) | 2,599  (51.18) | 2.495  (49.14) |  | 2,421  (47.68) | 2,586  (50.93) | 2,524  (49.70) | 2,548  (50.18) | 2,642  (52.04) |  |  |
|  | High level | 2,095  (41.26) | 2,014  (39.66) | 1,984  (39.17) | 1,979  (38.97) | | 1,950  (38.41) |  | 2,169  (42.71) | 1,948  (38.36) | 1,901  (37.44) | 1,942  (38.24) | 2,062  (40.61) |  | 2,147  (42.28) | 1,955  (38.50) | 2,036  (40.09) | 1,982  (39.03) | 1,902  (37.46) |  |  |
| Body Mass Index (BMI) (kg/m^2^) | | 23 (4) | 24 (4) | 24 (4) | 25 (4) | | 25 (4) | <0.001 | 23 (4) | 24 (4) | 24 (4) | 25 (4) | 25 (4) | <0.001 | 23 (4) | 24 (4) | 24 (4) | 25 (4) | 25 (4) | <0.001 |  |
| Alcohol (g/d) | | 11 (13) | 9 (10) | 9 (10) | 9 (9) | | 7 (9) | <0.001 | 8 (11) | 8 (10) | 9 (11) | 10 (10) | 10 (11) | <0.001 | 10 (12) | 10 (11) | 9 (10) | 8 (9) | 8 (9) | <0.001 |  |
| Smoking  status (%) | Never smoked | 2,830  (55.73) | 3,011  (59.29) | 2,987  (58.82) | 2,997  (59.02) | | 2,945  (58.01) | 0.004 | 2,897  (57.05) | 3,024  (59.55) | 3,012  (59.31) | 2,985  (58.78) | 2,852  (56.17) | <0.001 | 2,725  (53.66) | 2,846  (56.05) | 3,017  (59.41) | 3,101  (61.07) | 3,081  (60.69) | <0.001 |  |
|  | Ex-smoker | 1,637  (32.24) | 1,550  (30.52) | 1,522  (29.97) | 1,549  (30.50) | | 1,552  (30.57) |  | 1,693  (33.34) | 1,526  (30.05) | 1,524  (30.01) | 1,512  (29.78) | 1,555  (30.63) |  | 1,733  (34.13) | 1,611  (31.73) | 1,498  (29.50) | 1,447  (28.50) | 1,521  (29.96) |  |  |
|  | Current smoker | 611  (12.03) | 517  (10.18) | 569  (11.21) | 532  (10.48) | | 580  (11.42) |  | 488  (9.61) | 528  (10.40) | 542  (10.67) | 581  (11.44) | 670  (13.20) |  | 620  (12.21) | 621  (12.23) | 563  (11.09) | 530  (10.44) | 475  (9.36) |  |  |

Supplementary Table 7. Associations between nutrient intake and risk of multimorbidity with different levels using multinomial logistic regression analysis*.

| **Nutrient intake** | **Risk Ratio (95% CI)** | | **Risk Ratio (95% CI)** | |
| --- | --- | --- | --- | --- |
|  | **Low level**  **(CCI=1, N=4,944, 19.5%)** | ***p*** | **Moderate to High level (CCI>1,**  **N=2,855, 11.3%)** | ***p*** |
| Energy intake | 1.04 (1.01, 1.08) | 0.013 | 1.06 (1.02, 1.11) | 0.002 |
| Protein | 1.02 (0.99, 1.06) | 0.194 | 1.02 (0.98, 1.07) | 0.311 |
| Carbohydrate | 0.99 (0.95, 1.02) | 0.482 | 0.98 (0.94, 1.03) | 0.446 |
| Fat | 1.00 (0.96, 1.03) | 0.818 | 1.00 (0.96, 1.04) | 0.996 |
| SFAs | 1.00 (0.97, 1.03) | 0.995 | 0.99 (0.95, 1.03) | 0.665 |
| PUFAs | 1.01 (0.97, 1.04) | 0.768 | 1.02 (0.98, 1.06) | 0.375 |
| MUFAs | 0.99 (0.96, 1.03) | 0.697 | 1.00 (0.96, 1.04) | 0.944 |
| Vitamin C | 0.98 (0.95, 1.02) | 0.295 | 0.96 (0.92, 1.00) | 0.074 |
| Vitamin B1 | 1.02 (0.98, 1.05) | 0.354 | 1.00 (0.96, 1.05) | 0.922 |
| Vitamin B2 | 1.01 (0.97, 1.04) | 0.692 | 1.01 (0.97,1.06) | 0.531 |
| Vitamin B6 | 1.02 (0.98, 1.05) | 0.321 | 1.00 (0.96,1.04) | 0.962 |
| Vitamin B12 | 1.03 (0.99, 1.06) | 0.131 | 1.02 (0.98, 1.06) | 0.336 |
| Folate | 1.02 (0.99 ,1.06) | 0.226 | 1.02 (0.98, 1.06) | 0.383 |
| Vitamin A | 1.01 (0.98, 1.04) | 0.571 | 1.00 (0.96, 1.04) | 0.933 |
| Vitamin D | 1.03 (0.99, 1.06) | 0.124 | 1.04 (0.99, 1.08) | 0.093 |
| Vitamin E | 0.99 (0.96, 1.02) | 0.577 | 1.00 (0.96, 1.05) | 0.820 |
| Calcium | 1.00 (0.97, 1.04) | 0.800 | 0.97 (0.93, 1.02) | 0.233 |
| Iron | 0.97 (0.93, 1.00) | 0.050 | 1.01 (0.97, 1.06) | 0.498 |
| Zinc | 1.00 (0.97, 1.04) | 0.885 | 1.01 (0.97, 1.05) | 0.769 |

*Taking without multimorbidity (CCI=0, N=17,590, 69.3%) as the reference.
